# BRCA1 and BRCA2 deficient tumour models generate distinct ovarian tumour microenvironments and differential responses to therapy

**DOI:** 10.1186/s13048-023-01313-z

**Published:** 2023-11-28

**Authors:** Salar Farokhi Boroujeni, Galaxia Rodriguez, Kristianne Galpin, Edward Yakubovich, Humaira Murshed, Dalia Ibrahim, Sara Asif, Barbara C. Vanderhyden

**Affiliations:** 1 Cancer Therapeutics Program, Ottawa Hospital Research Institute, 501 Smyth Road, Ottawa, ON K1H 8L6 Canada; 2 Department of Cellular and Molecular Medicine, University of Ottawa, 451 Smyth Road, Ottawa, ON K1H 8M5 Canada; 3 Department of Biochemistry, Microbiology and Immunology, University of Ottawa, 451 Smyth Road, Ottawa, ON K1H 8M5 Canada

**Keywords:** *BRCA* mutations, Ovarian cancer, PARP inhibitors, Immune checkpoint inhibitors

## Abstract

Clinical trials are currently exploring combinations of PARP inhibitors and immunotherapies for the treatment of ovarian cancer, but their effects on the ovarian tumour microenvironment (TME) remain unclear. Here, we investigate how olaparib, PD-L1 monoclonal antibodies, and their combination can influence TME composition and survival of tumour-bearing mice. We further explored how *BRCA* deficiencies can influence the response to therapy. Olaparib and combination therapies similarly improved the median survival of *Brca1*- and *Brca2*-deficient tumour-bearing mice. Anti-PD-L1 monotherapy improved the survival of mice with *Brca1-*null tumours, but not *Brca2-*null tumours. A detailed analysis of the TME revealed that olaparib monotherapy resulted in a large number of immunosuppressive and immunomodulatory effects in the more inflamed *Brca1-*deficient TME but not *Brca2-*deficient tumours. Anti-PD-L1 treatment was mostly immunosuppressive, resulting in a systemic reduction of cytokines and a compensatory increase in PD-L1 expression. The results of the combination therapy generally resembled the effects of one or both of the monotherapies, along with unique changes observed in certain immune populations. *In-silico* analysis of RNA-seq data also revealed numerous differences between *Brca-*deficient tumour models, such as the expression of genes involved in inflammation, angiogenesis and PD-L1 expression. In summary, these findings shed light on the influence of novel therapeutics and *BRCA* mutations on the ovarian TME.

**Supplementary Information:**

The online version contains supplementary material available at 10.1186/s13048-023-01313-z.

## Background

Ovarian cancer refers to a group of malignancies that form primary tumours in the ovarian tissue [1]. This disease is the leading cause of death among gynecological malignancies, and it is the fifth leading cause of cancer-related death in women [2]. Absence of early-stage disease-specific symptoms, lack of effective screening tools, and high rates of relapse following initial treatment all contribute to the poor prognosis of ovarian cancer [3, 4]. Treatment is generally dependent on the pathological stage of the tumour and routinely involves cytoreductive surgery and chemotherapy [5, 6]. Depending on the cancer genotype and presence of mutations in DNA repair pathways such as the Breast Cancer-Associated genes (*BRCA*) 1 and 2, however, patients can also receive poly (ADP)-ribose polymerase inhibitors (PARPi) as maintenance therapy [7, 8].

The *BRCA* genes were first identified in 1990, and mutations in these genes were linked with the development of breast cancer [9]. BRCA1 and BRCA2 are central constituents of homologous recombination (HR), a mechanism through which cells repair double-stranded DNA breaks (DSBs) [10–13]. As a result of nonsense mutations in *BRCA1* and *BRCA2*, DSB repair cannot efficiently take place, and this results in continuous genome instability, high mutational load, chromosomal rearrangements, and overall defective genome maintenance, which results in cancer development [14]. Although initially useful in allowing a cancer cell to survive, continuous accumulation of mutations can result in cell death, even in cancer cells. As such, *BRCA-*mutant cells have to rely on alternative single-stranded break (SSB) DNA repair mechanisms. A prime example of SSB repair is the PARP repair pathway and the high reliance on this pathway allows for the survival of HR deficient (HRD) ovarian cancer cells [15]. However, the over-reliance on this singular repair pathway makes *BRCA*-mutated ovarian cancer cells susceptible to PARPi such as olaparib, which are now commonly employed as maintenance therapy [7, 16]. Ongoing clinical trials are also testing the efficacy of combining PARPi with immunotherapies such as PD-L1/PD-1 antibodies (NCT02953457).

Clinically, *BRCA* mutations have been simply regarded as HR defects, and *BRCA1-* and *BRCA2-*mutated ovarian cancers have been treated as the same disease. However, patients with *BRCA1* mutations tend to have a higher risk of developing ovarian cancer by the age of 80; 44% (95% C.I, 36%-53%) for *BRCA1* and 17% (95% C.I, 11%-25%) for *BRCA2* [17]. Similarly, the 5-year survival rate is shorter for patients with *BRCA1-*mutated tumours [18]. Such differences point to the possibility that these proteins are involved in pathways other than HR, and therefore can potentially influence the composition of the tumour microenvironment (TME). In fact, in preclinical models, mutations in *Brca1* and *Brca2* have been shown to influence response to immune checkpoint inhibitors (ICIs), with anti-PD-l blockade only improving survival of mice harbouring *Brca1*-null tumours [19]. Neoantigen presentation as a result of *BRCA*-mediated changes in mutational landscapes can also influence the infiltration of tumour-infiltrating lymphocytes (TILs) in the TME. For instance, patient-derived *BRCA*-mutated ovarian TMEs have a higher abundance of CD3 + and CD4 + T cells, as well as enhanced expression of immune checkpoint molecules [20]. However, the changes resulting in the ovarian TME as a consequence of both specific HR mutations and exposure to therapeutics such as PARPi remain largely unknown.

In this study, we used pre-clinical models to define the characteristics of the ovarian TME in response to the administration of PARPi and ICIs, which could ultimately lead to designing more effective combination therapies. In light of recent evidence that indicates the potential differences in *BRCA1* vs. *BRCA2-*mutated cancers, we further tested if tumours with *Brca1* and *Brca2* deficiencies respond differently to treatment by analyzing how the *Brca* status of ovarian tumours influence the TME composition and response to therapy.

## Results

### Olaparib increases DNA damage and reduces viability in *Brca-*deficient cell lines

**Fig. 1:**
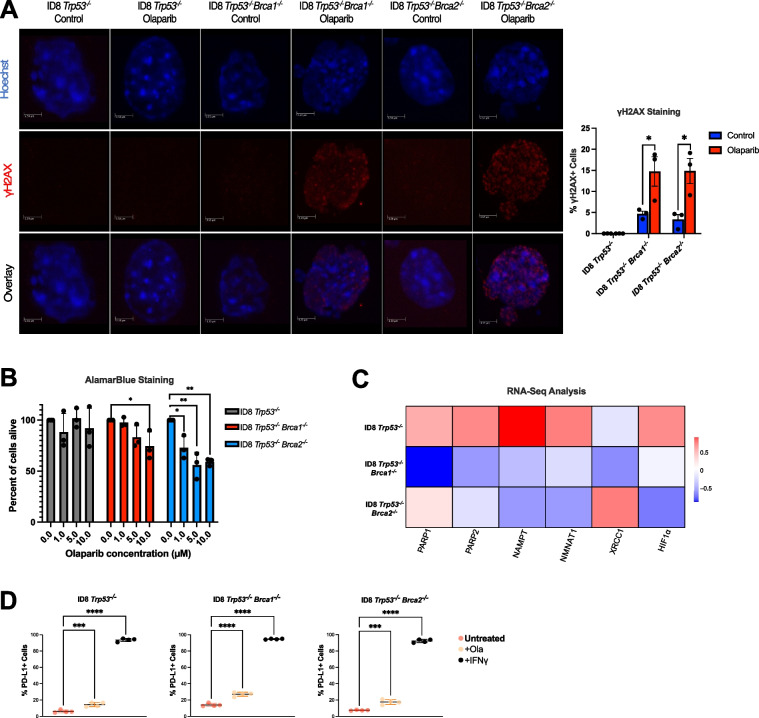
In vitro olaparib treatment induces DNA damage, reduces cancer cell viability and upregulates PD-L1. **A** Immunofluorescence staining images (left) and quantification (right) of γH2AX following a 24-h treatment of ID8 cells with 10 µM olaparib, representative of the extent of DNA damage. Student *t* test; *p < 0.05. **B** Histograms show the percent viability of ID8 cancer cells following treatment with 0, 1.0, 5.0 and 10 µM olaparib for 24 h, as assessed by AlamarBlue assays. Viability at each dose was compared to the 0 µM group. Analysis was done using a one-way ANOVA with Tukey’s multiple comparisons. Mean ± SEM (*n* = 3); *p < 0.05, **p < 0.01. **C** Heatmap presenting the expression levels of proteins involved in the PARP DNA repair pathway in the ID8 cell lines. RNA-seq data [23] collected from cell lines growing in vitro were analyzed to develop a normal distribution model of the expression of genes of interest. Each box represents the mean value of log-normalized RNA-seq data for 3 biological replicates. **D** PD-L1 expression in ID8 cells treated with 10 µM olaparib for 24 h, assessed using flow cytometry. Mean ± SEM (*n* = 3), one-way ANOVA; ***p < 0.0001, ****p < 0.01

The negative impact of PARP inhibition on the survival of ovarian cancer cells has been previously established [7, 8]. In this study, ID8 cells with CRISPR-mediated loss of *Trp53* alone or in combination with *Brca1* or *Brca2* deletion were used [21, 22]. To determine the extent of DNA damage and subsequent cell death in these models, we treated these cell lines with olaparib for 24 h and measured viability and γH2AX expression levels (Fig. 1A, B). In both *Brca*-deficient models, treatment with olaparib resulted in a significant increase in γH2AX staining, which was undetectable in the *Brca*-proficient control cells (Fig. 1A, left panel). Viability of the ID8 *Trp53*^*−/−*^ cells also did not change in response to any dose of olaparib (Fig. 1B). In contrast, cells with double knockout of *Trp53*^*−/−*^ and *Brca1*^*−/−*^ modestly responded to olaparib toxicity, with the highest dose of olaparib (10 µM) causing a significant 25% reduction in viability. Despite an equivalent amount of DNA damage, the ID8 *Trp53*^*−/−*^* Brca2*^*−/−*^ cells were the more olaparib-sensitive cell line, with viability reduced significantly at every dose of olaparib tested. Since olaparib differently affected *Brca*-null cell viability, the expression of genes associated with the PARP pathway in the ID8 cell lines was compared by querying RNA-seq data previously published in our lab [23]. We discovered that the ID8 *Trp53*^*−/−*^* Brca2*^*−/−*^ cells growing in vitro demonstrated a higher expression of both PARP1 and PARP2 compared to the *Brca1*^*−/−*^ model (Fig. 1C). Furthermore, expression levels of the DNA repair gene XRCC1 were highest in the *Brca2*-null model (Fig. 1C). Interestingly, nearly all PARP pathway-associated genes were highest in the *Brca*-proficient ID8 *Trp53*^*−/−*^ model. However, expression of XRCC1, the enzyme responsible for the actual DNA repair was low in relation to all other genes in the *Trp53*^*−/−*^ cells (Fig. 1C). Thus, in the ID8 model, mutations in the *Brca* genes are associated with reduced cell viability and increased susceptibility to DNA damage in response to olaparib treatment.

### Olaparib upregulates the expression of PD-L1

Previously, it was noted that in ovarian cancer cells, the PARP inhibitor niraparib can upregulate the expression of PD-L1, a critical regulator of immune activity [24]. To establish the influence of olaparib treatment on PD-L1 expression in the various ID8 models specifically, we measured its expression after a 24-h treatment. Regardless of the cell genotype, olaparib significantly increased the percentage of cells which expressed PD-L1 on their surface within the treated group, albeit to a proportion less than in the positive control, IFN-γ (Fig. 1D). The results of this experiment are analogous to the data found in the literature and identify PD-L1 as a target for combination therapy in vivo [24].

### Olaparib, anti-PD-L1 and their combination differently influence the survival of tumour-bearing mice

**Fig. 2:**
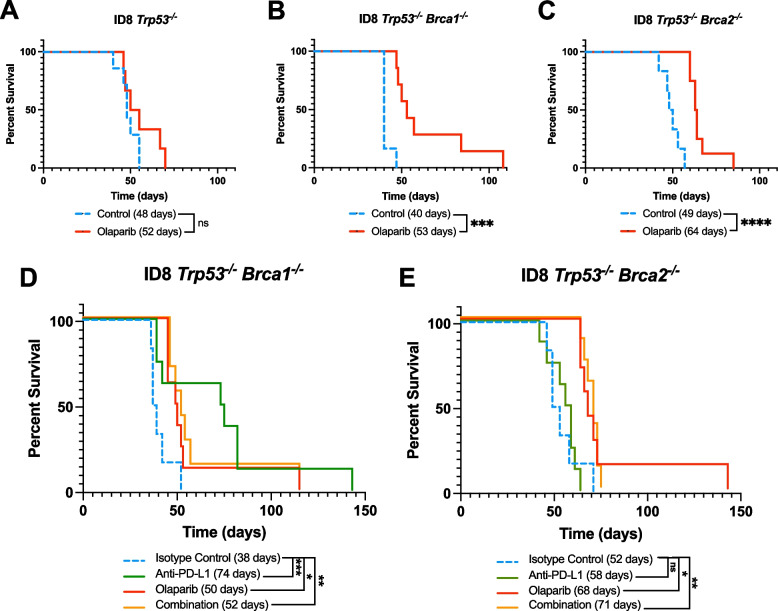
Syngeneic models of ovarian cancer differentially respond to treatment based on their genotype. 5 × 10^6^ ID8 *Trp53-/-* cells with or without *Brca1* or *Brca2* deficiency were injected IP to make syngeneic tumour models that were treated with olaparib, anti-PD-L1 or both. Olaparib was given by 18 daily IP injections at a dose of 50 mg/kg/day; the anti-PD-L1 was administered as five daily IP injections of 200 µg anti-PD-L1 followed by one 100 µg injection every four days for a total of 11 doses. The combination group received both drugs. Mice were euthanized at humane endpoint. **A**-**C** Kaplan–Meier survival plots showing the response of all three ID8 models to olaparib monotherapy relative to vehicle control. **D**-**E** Kaplan–Meier survival plots showing the response of *Brca1-* and *Brca2-*deficient ID8 models to olaparib, anti-PD-L1 and a combination of both drugs. Log-rank (Mantel-Cox) test. ns: not significant, *p < 0.05, **p < 0.01, ***p < 0.001

To assess the impact of treatments on the survival of tumour-bearing mice, two animal studies were performed. In the first study, mice were injected with one of the three ovarian cancer cell lines: ID8 *Trp53*^*−/−*^*,* ID8 *Trp53*^*−/−*^* Brca1*^*−/−*^ or ID8 *Trp53*^*−/−*^* Brca2*^*−/−*^. Treatment with olaparib did not significantly prolong the survival of the *Trp53*^*−/−*^ model, and the median length of survival of the control (48 days) and olaparib groups (52 days) were similar (Fig. 2A). In contrast, treatment significantly improved survival of the mice in the *Trp53*^*−/−*^*Brca1*^*−/−*^ and *Trp53*^*−/−*^*Brca2*^*−/−*^ models by approximately 33% and 30%, respectively (Fig. 2B, C). The absence of any response in the *Trp53*^*−/−*^ model resulted in the exclusion of this group from the next in vivo study.

As we had detected enhanced expression of PD-L1 in response to in vitro olaparib therapy, in the second animal study, mice harbouring ID8 *Trp53*^*−/−*^* Brca1*^*−/−*^ or ID8 *Trp53*^*−/−*^* Brca2*^*−/−*^ tumours were treated with either olaparib or anti-PD-L1 monotherapies, or a combination of both drugs, using the treatment regimen summarized in Supplementary Figure S 1. In the *Trp53*^*−/−*^* Brca1*^*−/−*^ model, the anti-PD-L1 monotherapy nearly doubled the median survival of tumour-bearing mice (74 days) compared to the isotype control (38 days), making it the most beneficial overall treatment in terms of survival (Fig. 2D). The survival of mice given olaparib monotherapy (50 days) and combination therapy (52 days) were very similar and were both significantly longer than the isotype control (Fig. 2D). In the *Trp53*^*−/−*^* Brca2*^*−/−*^ model, the anti-PD-L1 monotherapy did not improve survival (Fig. 2E). However, the olaparib monotherapy did significantly improve the survival of mice harbouring *Brca2*-deficient tumours (68 vs. 52 days; Fig. 2E). Similar to the *Brca1*-deficient group, the outcomes from the combination therapy were not different from the survival after olaparib monotherapy (Fig. 2E). Overall, the olaparib and combination therapy similarly improved the survival of both models, but no synergy was observed. However, the anti-PD-L1 monotherapy demonstrated efficacy specifically in prolonging the survival of the *Trp53-/- Brca1-/-* mice, while its effects on other treatment groups were not significant.

### Olaparib, anti-PD-L1 and their combination transform the immune composition of the *Brca*1- and *Brca2*-deficient tumour microenvironments

The effects of PARPi treatment on the composition of the ovarian TME have not been studied extensively. As such, we set out to characterize the immune composition of various innate and adaptive immune cell populations within the PARPi-treated animals 36 h after the end of therapy. We collected ascites fluid, or performed a peritoneal wash, as well as spleen and analyzed the immune cell populations by flow cytometry (Supplementary Fig. S2). We also characterized anti-PD-L1 as a monotherapy and in combination with olaparib. This addition provided us with a more sophisticated and complete image of the TME composition in response to novel therapeutics. Analysis of the immune composition of the spleen from tumour-bearing mice was also performed to determine the influence of these therapeutics on the systemic immune system (Supplementary Fig. S 3-S 4).

**Fig. 3:**
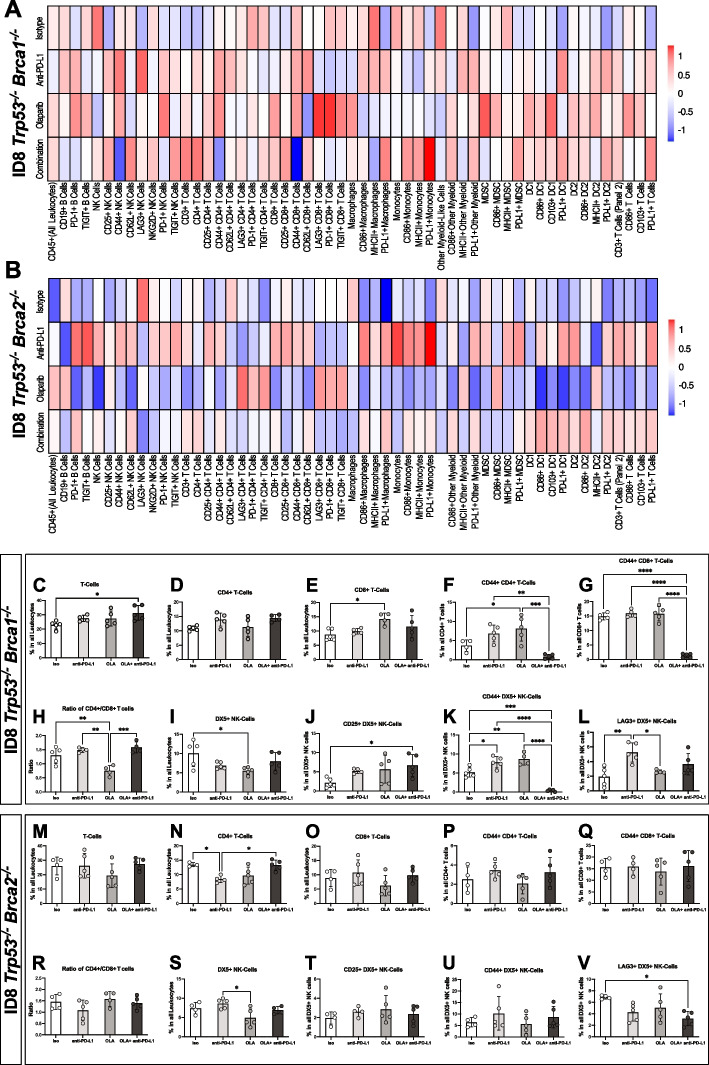
*Brca* deficiencies cause differential changes in the tumour microenvironment in response to therapy. **A**-**B** Heatmaps presenting the relative abundance of immune cell types in the (**A**) ID8 *Trp53*^*−/−*^* Brca1*^*−/−*^ and (**B**) *Trp53*^*−/−*^* Brca2*^*−/−*^ tumour microenvironments. Peritoneal washes (*n* = 5 mice per group) were collected from tumour-bearing mice that had been treated with olaparib, anti-PD-L1 or their combination, and analyzed by flow cytometry 36 h after the last treatment. For each cell type, the value was calculated as the percent of all leukocytes. The percentages were normalized, and each box represents the mean of the 5 biological replicates. **C**-**V**. Histograms showing the immune populations that are significantly different from isotype control. The significant changes in cell populations are divided based on genotype: (**C**-**L**) for ID8 *Trp53*^*−/−*^* Brca1*^*−/−*^ and (**M-V**) for the *Trp53*^*−/−*^* Brca2*.^*−/−*^ tumour-bearing mice. Each dot represents one biological replicate. Mean values with SEM are shown. ISO = isotype control group and OLA = olaparib treated group. One-way ANOVA with Tukey’s multiple comparison test; *p < 0.05, **p < 0.01, ***p < 0.001, ****p < 0.0001

Heatmaps summarize the characterization of the peritoneal microenvironment of the ID8 *Trp53*^*−/−*^* Brca1*^*−/−*^ (Fig. 3A) and *Trp53*^*−/−*^* Brca2*^*−/−*^ (Fig. 3B) models. All three treatments uniquely transformed the *Trp53*^*−/−*^* Brca1*^*−/−*^ TME (Fig. 3A). The administration of monotherapies and their combination was associated with higher percentages of multiple different cell types as seen in the positive z-scores in the heatmap rows which represent treatment groups (Fig. 3A). The cell frequency changes which are significantly different from isotype control and/or the combination therapy are shown in Fig. 3C-L and included some notable effects. The combination therapy resulted in an 8% increase in overall T cell frequency compared to the isotype control (Fig. 3C). The olaparib monotherapy increased the total frequency of CD8 T cells and activated (CD44 expressing) CD4 T cells by approximately 5%, thereby doubling this population (Fig. 3E, F). Exceptionally, the combination therapy significantly reduced the frequency of activated, CD44 + CD4 + T cells to near zero levels compared to the monotherapies (Fig. 3F). Similarly, the combination therapy resulted in a > 90% reduction in activated, CD44 + CD8 + T cells compared to all other groups, reducing the percentage of these cells to near zero levels (Fig. 3G). Olaparib alone also reduced the CD4/CD8 T cell ratio compared to all groups by approximately 50% (Fig. 3H).

The therapies also uniquely influenced the composition of NK cell populations. The olaparib monotherapy reduced the overall NK cell frequency by ~ 50% (Fig. 3I) while the combination therapy significantly enhanced CD25 + NK populations (Fig. 3J). The monotherapies both increased the frequency of CD44 + NK cells, while the combination therapy reduced this population to nearly zero (Fig. 3K). Lastly, the anti-PD-L1 monotherapy doubled the population of LAG3 positive NK cells (Fig. 3L).

In contrast to the *Brca1*-null TME, the treatment of *Brca2*-null tumour-bearing mice resulted in fewer significant changes in the immune cell populations found in the TME, as shown in the heatmap in Fig. 3B. The TME derived from *Brca2*-null group seem to exhibit a higher prevalence of negative z-scores, especially in response to olaparib alone (Fig. 3B). While the vast majority of immune cell populations appear to be lowest in the TME of the olaparib group, some cell types are present at higher proportions, namely those expressing LAG3, PD-1 and TIGIT, all of which are markers of exhaustion and/or inhibition (Fig. 3B). Statistical analysis of the various cell types revealed a small number of significant differences between the isotype control and treatment groups (Fig. 3N-V). The percentage of LAG3 + NK cells was reduced to about half by the combination treatment, relative to the isotype control (Fig. 3V). The anti-PD-L1 monotherapy reduced the CD4 + T cell population by ~ 50% (Fig. 3N). Overall, responses to therapy varies substantially between these models. The immune cell composition of the *Brca2*-null TME revealed much fewer modifications than those seen in the *Brca1*-null model, suggesting the absence or poor efficacy of the anti-PD-L1 monotherapy in the *Brca2*-null model may be due to a lack of antitumoral immune stimulation in the TME.

**Fig. 4:**
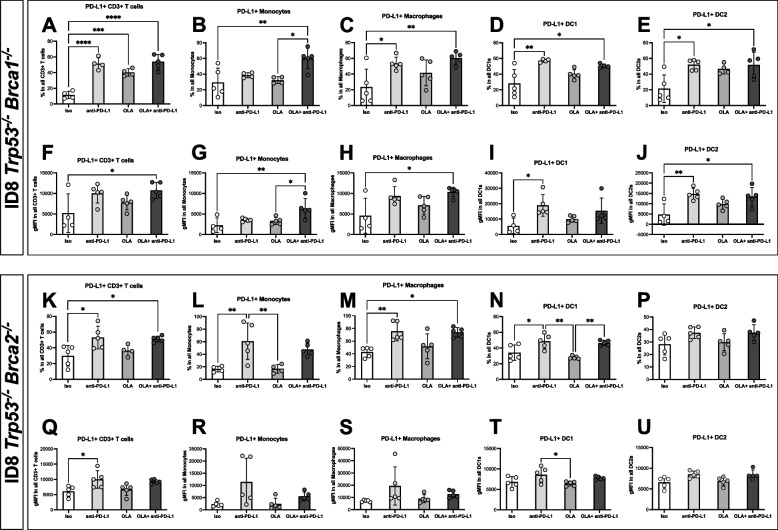
In vivo treatment with anti-PD-L1 results in a compensatory upregulation of this marker in both *Brca-*deficient tumour models. Peritoneal washes (*n* = 5 mice per group) were collected from tumour-bearing mice that had been treated with olaparib, anti-PD-L1 or their combination, and analyzed by flow cytometry 36 h after the last treatment. Only the immune populations which are significantly different from isotype control or the combination therapy are shown. The immune populations were divided based on genotype: (**A**-**J**) for ID8 *Trp53*^*−/−*^* Brca1*^*−/−*^ and (**K**-**U**) for *Trp53*^*−/−*^* Brca2*.^*−/−*^ tumours. Each dot represents one biological replicate. Mean values with SEM are shown. ISO = isotype control group and OLA = olaparib treated group. One-way ANOVA with Tukey’s multiple comparison test; *p < 0.05, **p < 0.01, ***p < 0.001, ****p < 0.0001

Surprisingly, in both the *Brca1-* and *Brca2-*deficient tumour models used in this study, the in vivo treatment with anti-PD-L1, whether as a monotherapy or in combination with olaparib, resulted in a systemic increase in nearly all the studied immune cells presenting surface PD-L1. Both the expression levels of this marker and the percentage of cells expressing it were amplified significantly (Fig. 4A-U). This systemic increase was one of the few changes that were consistently changed in all tissues analyzed in this study. Taken together, in vivo treatment with anti-PD-L1 results in upregulation of PD-L1 in the ovarian tumour microenvironment.

### In vivo treatments differentially influence the cytokine composition of the ascites fluids in the *Brca*-deficient models

**Fig. 5:**
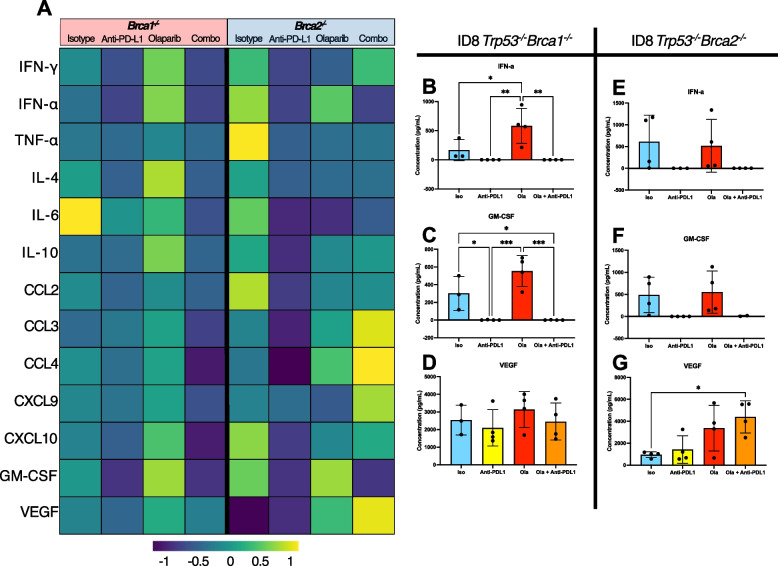
Cytokine/chemokine concentrations in ascites reflect BRCA-dependent differences in response to treatment. Ascites fluids were collected at humane endpoint (n = 4 per group). Cytokines were quantified using the LEGENDplex Mouse Cytokine Release Syndrome flow-based assay. **A** Heatmap showing the normalized concentrations of cytokines in the ID8 *Trp53*^*−/−*^* Brca1*^*−/−*^ and ID8 *Trp53*^*−/−*^* Brca2*^*−/−*^ models. Each box represents the mean of 4 replicates. **B**-**G** Histograms indicating the concentrations that are significantly different from isotype control or the combination in at least one model. The histograms are divided based on tumour genotype: (**B**-**D**) shows data from the ID8 *Trp53*^*−/−*^* Brca1*^*−/−*^ ascites and (**E**–**G**) the *Trp53*^*−/−*^* Brca2*.^*−/−*^ ascites fluid. Each dot represents the ascites supernatant from one biological replicate. Mean values with SD are shown. Analysis was done using a one-way ANOVA with Tukey’s multiple comparison; *p < 0.05, **p < 0.01, ***p < 0.001

The knowledge of the effects of PARPi and monoclonal antibody therapies on cytokine production in the ovarian TME is minimal. As such, we set out to determine the effects of these treatments on the concentrations of 13 cytokines and chemokines. The cytokine arrays conducted on ascites fluid collected at humane endpoint revealed several changes, as shown in the heatmap in Fig. 5A. Of those, changes that were significant in either the *Brca1* or *Brca2*-null models are individually presented in Fig. 5B-G.

As presented in the heatmap, the anti-PD-L1 monotherapy resulted in a general reduction in cytokine concentrations in the ID8 *Trp53*^*−/−*^* Brca1*^*−/−*^ model compared to isotype control (Fig. 5A). Olaparib monotherapy, on the other hand, resulted in cytokine concentrations that were often higher than the isotype control (Fig. 5A). Changes in cytokine production in the ID8 *Trp53*^*−/−*^* Brca2*^*−/−*^ model in response to anti-PD-L1 were akin to those in the *Brca1*^*−/−*^ model, but with more substantial reductions (Fig. 5A). Unlike the *Trp53*^*−/−*^* Brca1*^*−/−*^ model, olaparib had a general suppressive effect on the levels of several cytokines in the *Brca2*^*−/−*^ model (Fig. 5A). The combination therapy had similar effects, leading to both increases and decreases in cytokine production that followed the same trends as the olaparib monotherapy (Fig. 5A).

It is notable that some changes in cytokine abundance were consistent across both models, while others were only present in one model (Fig. 5B-G). Compared to all other cytokines in the *Brca1*-null TME, IFN-*a* production was significantly higher with olaparib monotherapy (Fig. 5B). Remarkably, treatment with anti-PD-L1 on its own or in combination with olaparib drastically reduced IFN-*a* concentrations to near undetectable levels (Fig. 5B). These differences were also seen in the *Brca2*-null model (Fig. 5E). Expression of granulocyte–macrophage colony-stimulating factor (GM-CSF) closely resembled that of IFN-*a*, as anti-PD-L1 treatment completely diminished GM-CSF production in both models (Fig. 5C, F), however these changes were only significant in the *Brca1*-null group (Fig. 5C).

A synergistic effect of both treatments resulted in the highest VEGF concentration after combination therapy of the *Brca2-*null model (Fig. 5G), as the concentrations of VEGF were more than 4 times greater than the isotype control. Conversely, there were no changes VEGF concentrations of the *Brca1-*null TMEs (Fig. 5D). In summary, the impact of therapy on cytokine concentrations within the ovarian TME was found to vary between treatments, with anti-PD-L1 exhibiting a unique suppressive effect. In contrast, cytokine concentrations in the olaparib-treated group were generally similar to or higher than the isotype control, albeit not always significantly different. Furthermore, akin to the variations in the cellular composition of the TME, the alterations in the cytokine profiles were found to differ greatly between the *Brca1*- and *Brca2*-null models.

### Depletion of* Brca1 *and *Brca2* in ovarian cancer cells differentially influences the expression of genes involved in the TME composition

**Fig. 6:**
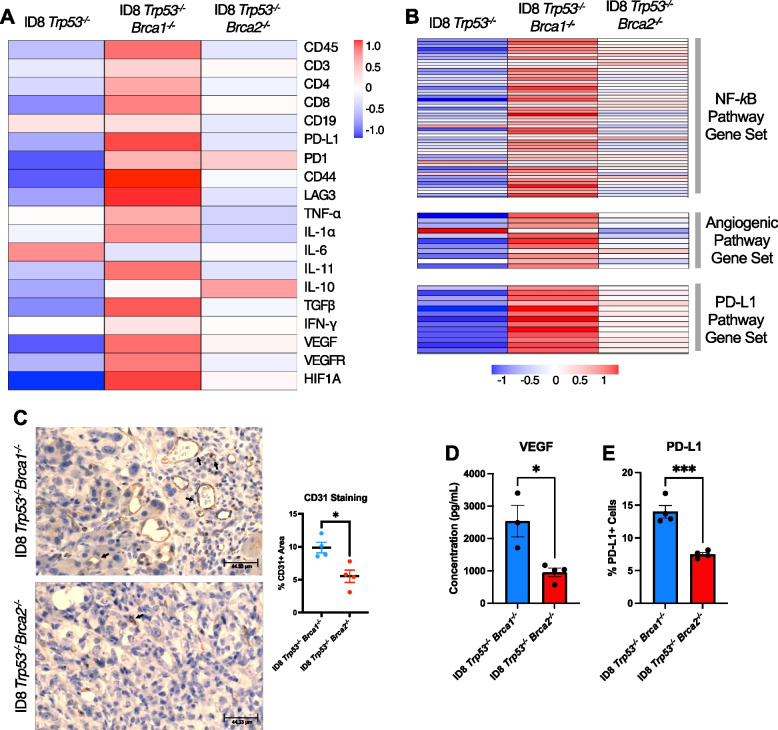
Deficiencies in *Brca1* and *Brca2* differently influence the expression of immune-related factors in the ovarian tumour microenvironment. **A** Heatmap showing the relative expression of immune-related genes in intraperitoneal tumours. Each box represents the mean value of log-normalized bulk RNA-seq data from 4–6 biological replicates. Gene names were replaced by the corresponding protein name. **B** Pathway analyses for three gene sets that demonstrate the activities of the NF-*k*B, angiogenic and PD-L1 pathways. The activity of the pathway was approximated by measuring the expression of 12–53 genes which are known to be downstream for the activity of each protein of interest. **C** Immunohistochemical staining images (left) and quantification of CD31^+^ cells (brown) in *Brca-*deficient tumours collected at the humane endpoint (*n* = 4) from the mice in the isotype control group. Quantifications are based on the percentage area of CD31^+^ cells in relation to all nucleated cells. **D** Histograms presenting the concentrations of VEGF in the ascites fluid collected from mice harbouring *Brca1-* and *Brca2-*deficient tumours. Cytokines were quantified using the LEGENDplex Mouse Cytokine Release Syndrome flow-based assay. **E** Histograms presenting the percentage of PD-L1^+^ cells in untreated ID8 *Trp53*^*−/−*^* Brca1*^*−/−*^ and ID8 *Trp53*^*−/−*^* Brca2*.^*−/−*^ cell lines. Expression levels were quantified using flow cytometry. Mean ± SEM are shown for all histograms. Analysis was done using the student *t* test; *p < 0.05, ***p < 0.001

As the TME analysis from the two models revealed differences in various cell populations and cytokines, we analyzed previously published bulk RNA sequencing data [23] from both *Brca-*deficient IP tumour models to further investigate the differences between them. The analysis revealed notable differences in the transcript levels of several cell type markers in *Brca-*null TMEs. Expression of CD45, the pan-leukocyte marker, was higher in *Trp53*^*−/−*^* Brca1*^*−/−*^ tumours compared to both the *Trp53*^*−/−*^* Brca2*^*−/−*^ and the *Brca-*proficient *Trp53*^*−/−*^ models (Fig. 6A). Similarly, expression levels for various immune cell markers such as CD3, CD4, and CD8 were highest in the *Brca1-*null tumours. Expression of genes associated with immune cell function also varied between the three models. For instance, markers of both leukocyte activation (CD44) and exhaustion (LAG3) were also highest in the *Brca1-*null tumours (Fig. 6A). Aside from cell type and functional markers, the expression of genes encoding cytokines differed between the models as well. For pro-inflammatory cytokines such as TNF-*a* and interleukin-1 alpha (IL-1*a*), expression levels were greatest in the ID8 *Trp53*^*−/−*^* Brca1*^*−/−*^ tumours, while the anti-inflammatory cytokine IL-10 was expressed at the lowest level. However, it must be noted that expression levels of IL-11, a potent anti-inflammatory cytokine, were also higher in the *Brca1*-null model (Fig. 6A).

Given the large number of differences detected in the immune compositions of the *Brca*-deficient TMEs, we investigated further the expression of a key inflammatory mediator, nuclear factor kappa-light-chain-enhancer of activated B cells (NF-*k*B) [25]. This was achieved by measuring the expression levels of a set of 53 genes that are known to be downstream of this transcription factor. NF-*k*B, in addition to being a primary driver of inflammation, is a key downstream constituent of one of the major driving forces in TME composition: cyclic GMP-AMP synthase-stimulator of interferon genes (cGAS-STING) signaling [26]. Therefore, analysis of NF-*k*B signaling could be used as an indicator of a potential change in cGAS-STING activity. Examination of this data revealed that NF-*k*B activity was highest in the *Trp53*^*−/−*^* Brca1*^*−/−*^ model, modest in the *Trp53*^*−/−*^* Brca2*^*−/−*^ tumours and lowest in the *Trp53*^*−/−*^ group (Fig. 6B). Similar pathway analyses were done for 12 genes associated with angiogenesis, such as *Vegf, Vegfr1, Cd31,* and *Hif1a.* Genes associated with angiogenesis were generally higher in the *Trp53*^*−/−*^* Brca1*^*−/−*^ tumours (Fig. 6B). These results were confirmed at the protein level using immunohistochemistry and cytokine arrays. Immunohistochemical staining for CD31 revealed that *Trp53*^*−/−*^* Brca1*^*−/−*^ tumours have greater levels of angiogenesis compared *Trp53*^*−/−*^* Brca2*^*−/−*^ tumours (Fig. 6C). Likewise, results from the ascites cytokine array revealed that concentrations of VEGF are significantly higher in the ascites of the *Trp53*^*−/−*^* Brca1*^*−/−*^ model compared to the *Trp53*^*−/−*^* Brca2*^*−/−*^ derived samples (Fig. 6D).

Lastly, a pathway analysis of 13 genes downstream of PD-L1 signaling was performed to measure PD-L1 activity in these models. The expression levels of all the genes analyzed for this pathway were once again consistently higher in the *Trp53*^*−/−*^* Brca1*^*−/−*^ tumour samples (Fig. 6B). Results of flow cytometry staining support these findings, as the baseline levels of PD-L1 positive cells were found to be significantly higher in the *Brca1-*deficient cancer cells in vitro when compared to *Trp53*^*−/−*^* Brca2*^*−/−*^ cells (Fig. 6E). Taken together, the RNA sequencing data with validations at the protein level by flow cytometry and immunohistochemistry provide strong evidence that, compared to the *Trp53*^*−/−*^* Brca2*^*−/−*^ model, the ID8 *Trp53*^*−/−*^* Brca1*^*−/−*^ tumours have a more “inflamed” TME, with increased composition of leukocytes, as well as higher expression of inflammatory cytokines and angiogenesis associated genes.

## Discussion

Despite the fact that they’ve been studied extensively, the effects of PARPi and PD-L1 monoclonal antibodies treatment on the composition of the ovarian TME remain unknown. In this study, we used syngeneic models of ovarian cancer to investigate the effects of therapy on the immune and cytokine profiles of the ovarian TME. Furthermore, we studied the role of deficiency of the *BRCA1/2* DNA repair genes in TME composition, response to therapy, and expression of genes involved in various signalling pathways.

In the initial in vitro analyses, the *Brca*-deficient ID8 models were differently sensitized to olaparib-induced toxicity, with the *Brca2*-deficient tumours demonstrating greater susceptibility to PARP inhibition. This dissimilarity in drug sensitivity between the *Brca1*- and *Brca2-*deficient cells may in part be explained by the expression of the PARP-pathway associated genes, the expression levels for which are higher in the ID8 *Trp53*^*−/−*^* Brca2*^*−/−*^ cells. Thus, the *Brca2*^*−/−*^ cell line may be more dependent on the PARP DNA repair pathway and more sensitive to PARPi-induced toxicity. Flow cytometry following *in* vitro treatment with olaparib further revealed an increase in PD-L1 expression regardless of the cell’s genotype. Previous studies have linked this upregulation to a PARPi-induced phosphorylation and subsequent inhibition of glycogen synthase kinase-3 beta (GSK3β), which under normal conditions results in phosphorylation-dependent degradation of PD-L1 [27, 28]. This observation allowed us to select PD-L1 as a target for the in vivo combination therapy.

When examining the impact of treatment on the survival of tumour-bearing mice, the olaparib monotherapy did not improve the survival of mice harbouring *Brca*-proficient tumours, as expected, as the cancer cells would have been able to readily repair their DNA using HR. As such, only the two *Brca-*deficient models of ovarian cancer were treated with olaparib, anti-PD-L1 or a combination of both drugs in the subsequent study. Olaparib improved the survival of both the *Brca1* and *Brca2-*deficient models by approximately 30%. Previous studies have noted similar improvements in survival of *Brca*-deficient models of ovarian cancer in response to PARPi treatment, although in different cell lines, mice strains and PARPi [29]. Although it resulted in the generation of a relatively cold TME, olaparib did not yield any statistically significant changes in the immune populations of *Trp53*^*−/−*^* Brca2*^*−/−*^ model.

In contrast, olaparib monotherapy resulted in a plethora of immunomodulatory changes in T and NK cell populations within the *Trp53*^*−/−*^* Brca1*^*−/−*^ TME. For instance, the population of CD8 + T cells nearly doubled in response to olaparib treatment. Due to their extraordinary antitumoral abilities, CD8 + cytotoxic T lymphocytes (CTLs) are the favoured adaptive immune cell for antitumour immunity [30]. In ovarian cancer, increased infiltration of TME by CD8 + CTL has been associated with better prognosis and survival [30–32]. Similarly, olaparib treatment increased the percentage of activated, CD44 + CD4 T cells in the *Brca1*^*−/−*^ TME. Activated CD4 + T cells have various antitumour capabilities, such as directly activating CD8 + T cells by IL-2 secretion or the production of cytokines such as TNF-*a* and IFN-γ that can further attract and activate various antitumoral immune cells [33–35]. Furthermore, olaparib reduced the ratio of CD4 + /CD8 + T cells in the ID8 *Trp53*^*−/−*^* Brca1*^*−/−*^ TME. In clinical studies, a lower ratio has been associated with superior outcomes in ovarian cancer patients [36, 37]. However, not all the associated changes within the *Brca1*^*−/−*^ TME were inflammatory, as olaparib treatment also resulted in reduced frequency of NK cells and enhanced PD-1 expression. NK cells directly contribute to the killing of cancer cells through the secretion of granzymes and perforin, and thus their inhibition through PD-1 is associated with weaker antitumour capabilities [38–40]. In order to separate the effects of PARP inhibition on immunomodulation from its effects on the DNA damage repair pathway in the *Trp53*^*−/−*^* Brca1*^*−/−*^ model, it would be valuable to test the treatment of these tumours with olaparib in immunodeficient mice.

Unlike the olaparib monotherapy, the survival benefits of anti-PD-L1 treatment were limited to the *Trp53*^*−/−*^* Brca1*^*−/−*^ model, where treatment doubled the length of survival. The prolonged survival may be due to a variety of factors that make this tumour model more susceptible to PD-L1 blockade. Firstly, the changes in the *Brca1*^*−/−*^ TME as a result of anti-PD-L1, such as greater expression of CD44 and LAG3 on NK cells, can improve the antitumour activity of this cell type. In contrast, aside from the systemic rise in PD-L1, the only statistically significant change in the *Brca2*^*−/−*^ TME was a reduction of CD4 + T cells. Furthermore, the RNA-seq pathway analysis and flow cytometry data revealed that the *Trp53*^*−/−*^* Brca1*^*−/−*^ tumours have higher levels of PD-L1 expression and thus have more abundant target sites for the drug. This aligns well with evidence indicating higher expression of this molecule in *BRCA1-*deficient tumours from breast and ovarian cancer patients [19, 41]. The specific increase in the *Brca1-*deficient models may be due to the intracellular role that PD-L1 plays in DNA repair. Recent evidence has emerged which identified PD-L1 as a translocator of BRCA1 from the cytoplasm into the nucleus [42]. As such, effective homologous recombination is potentially dependent on the intracellular activity of PD-L1. *Brca1-*null cells may be upregulating this protein in an attempt to increase translocation of absent BRCA1 from the cytoplasm into the nucleus.

Our results are in agreement with recent reports that the *Brca1*^*−/−*^ TMEs are more infiltrated by immune cells and are more immunogenic than the *Brca2*^*−/−*^ TMEs in humans [19, 43]. The higher expression of neoantigens as a result of greater tumour mutational burden in *BRCA*1-mutated tumours may in part account for the superior immune cell attraction and infiltration [19]. Greater abundance of immune cells within the *Trp53*^*−/−*^* Brca1*^*−/−*^ TME makes this tumour model ideal for anti-PD-L1 treatment, as the highly abundant immune cells will be able to continuously eliminate cancer cells without being inhibited [44, 45]. One other factor that might contribute to the greater abundance of TILs in *Trp53*^*−/−*^* Brca1*^*−/−*^ TMEs is enhanced angiogenesis, supported by the RNA-seq pathway analysis, as well as higher levels of CD31 and VEGF proteins in this model. While the mechanism underlying this increase in vascularity requires further investigation, one possibility may be associated with the ability of BRCA1 to bind to the active function-2 domain of estrogen receptor alpha (ER*-α*), thereby preventing its dimerization with estradiol [46]. The dimerized complex of ER-*α* and estradiol induces the transcription of many genes, including *VEGFA* [47]. As such, in the absence of BRCA, this complex can continually induce the expression of angiogenic proteins. Greater tumour mutational burden, neoantigen presentation, increased PD-L1 expression and immune cell infiltration may all combine to make the *Trp53*^*−/−*^* Brca1*^*−/−*^ model more responsive to anti-PD-L1 treatment.

The survival of mice treated with the combination therapy was nearly identical to the olaparib monotherapy in both models. This observation is reasonable for the *Trp53*^*−/−*^* Brca2*^*−/−*^ tumour-bearing mice, as this group did not respond to anti-PD-L1 treatment. As such, olaparib was hypothetically the only effective antitumoral agent in the combination mixture for this specific model. However, the *Trp53*^*−/−*^* Brca1*^*−/−*^ tumour-bearing mice responded remarkably well to both treatments and it was expected that there would be a synergistic increase in survival of the mice treated with the combination therapy. One plausible explanation for the lack of this synergy could be the unexpected reduction in CD44 expression in NK, CD4 + , and CD8 + cells within the TME of the *Trp53*^*−/−*^* Brca1*^*−/−*^ combination group. CD44 plays a role in regulating cell adhesion and migration of antigen experienced T cells, and CD44 ligation can augment T cell activation after antigen encounter and promote T cell survival [48]. Although the molecular mechanisms underlying this reduction are unclear, it is possible that the combination of the two monotherapies simply prohibited the activation of these immune cells which in turn prevented their immune-mediated cancer cell elimination. As such, the only effective antitumoral agent in this mixture would have been the olaparib which results in direct HRD cancer cell death.

Two notable observations in the analysis of the ascitic TME and spleen tissue from tumour-bearing mice were the systemic increase in PD-L1 expression on many immune cell types, as well as a universal ablation of both GM-CSF and IFN-*α* in response to anti-PD-L1. These changes were observed whether the PD-L1 antibody was given as a monotherapy or in combination with olaparib. The systemic increase in PD-L1 positivity in both models is likely a compensatory reaction to anti-PD-L1 blockade. A similar compensatory upregulation of various other immune checkpoint molecules has been noted in other studies [49–51]; however, here we demonstrate for the first time that cells within the ovarian TME can upregulate the expression of PD-L1 in response to anti-PD-L1 blockade. We hypothesize that the suppression of GM-CSF and IFN-*α* may be a downstream consequence of the systemic rise in PD-L1. A recent study by Hodgins et al. demonstrated that intracellular PD-L1 activity can reduce type I interferon production through metabolic alterations that increase glycolysis and reduce oxidative phosphorylation [52]. PD-L1 drives upregulation of the glycolytic pathway and results in the accumulation of pyruvate, which is converted into lactate, and cytoplasmic lactate buildup negatively regulates the production of type I interferons [52]. One possible mechanism for this inhibition is the lactate-induced inhibition of cGAS-STING signaling, a major driver of both type I interferons and NF-*k*B [26, 53]. A lactate-driven decrease in cGAS-STING activity could also explain the reduction in GM-CSF since cGAS-STING-induced NF-*k*B activity is known to regulate GM-CSF production by direct transcriptional activation [25].

In this study, analysis of the response to treatments in vitro and in vivo included TME characterization, cytokine arrays and RNA sequencing that revealed numerous differences between *Brca-*deficient tumour models of ovarian cancer. ID8 *Trp53*^*−/−*^* Brca1*^*−/−*^ tumours have a more “inflamed” phenotype, with greater abundance of TILs, higher expression of inflammatory genes and cytokines, as well as greater activity of inflammatory and angiogenic pathways. Clinically, *BRCA1-* and *BRCA2*-mutated ovarian cancers have been treated as the same disease. However, it is clear that these mutations can have significant and distinct impacts on patient outcomes. The findings of this study, as well as the ongoing clinical trials, can help to shed light on the influence of *BRCA* mutations on the response to treatment of ovarian cancer. Further study of the non-HR roles of the BRCA proteins is crucial for understanding the breadth of consequences resulting from their absence. By considering the specific *BRCA* status of the tumour, clinicians may be able to provide treatments that are personalized to the needs of patients with various genotypes.

## Methods

### Cell lines

The ID8 *Trp53*^*−/−*^, *Trp53*^*−/−*^* Brca1*^*−/−*^ and *Trp53*^*−/−*^* Brca2*^*−/−*^ were generously provided by Dr. Iain McNeish and were cultured using Dulbecco's Modified Eagle's Medium (DMEM) (Corning, #10–013-CV) supplemented with 4% Fetal Bovine Serum (FBS) (ThermoFisher, #12,483–020) and 1 × Insulin–Transferrin–Sodium-Selenium (Sigma-Aldrich Roche, #11,074,547,001) as previously described [21, 22, 54]. Prior to cancer cell injection in each animal study, mycoplasma testing was performed.

### In vitro olaparib treatment

In order to assess cancer cell viability in response to PARPi treatment, ID8 cells were treated with olaparib, and viability was assessed using AlamarBlue assays. ID8 cells were seeded in a 96-well plate at a density of 1.0 × 10^3^ cells/well 24 h prior to the start of treatment. A stock of olaparib (MedChemExpress; HY-10162) was prepared by dissolving the drug in dimethyl sulfoxide (DMSO) at a concentration 25.57 mM. Media with various concentrations of olaparib (0, 1, 5 and 10 µM) were prepared by dissolving the DMSO/olaparib stock mixture in DMEM media. The original culture media were then aspirated from the wells and replaced with media containing various concentrations of olaparib. After the 24-h treatment period, the medium was aspirated from each well and replaced with DMEM media containing 10% AlamarBlue cell viability reagent (ThermoFisher; #DAL1100). The plate was incubated in the dark at 37 °C for 3 h and then read using a Bio-Tek Microplate Reader for which the absorbance and emission wavelengths were set at 530 and 590 nm, respectively. The data collected from the treated cells were normalized to the untreated samples. The average results of three experiments each performed with three technical replicates were pooled for statistical analyses.

### Mouse models and in vivo studies

To assess the response of ovarian tumours to therapy, three different syngeneic models of intraperitoneal (IP) tumours were used and information regarding each model can be found in Supplementary Table S1. C57BL/6 mice (The Jackson Laboratory; #664) were used for syngeneic model development for all cell lines. All animals were housed in the Animal Care and Veterinary Services (ACVS) facilities at the University of Ottawa. The experimental protocols adhered to the standards defined by the guidelines of the Canadian Council on Animal Care and were approved by the University of Ottawa Animal Care Committee. The animals were provided with the standard chow diet and monitored on a daily basis. For tumour development, 8-week-old mice were given IP injections of 5.0 × 10^6^ cells diluted in 100 µL of phosphate-buffered saline (PBS) (ThermoFisher; #14,190–144). To allow a standardized period of time for tumours to establish before treatment, the treatments started 25% into the predicted length of survival of each tumour model, which was established in previous studies in the Vanderhyden lab [54] (Supplementary Table S1).

#### Olaparib in vivo study

In the first animal study, the ID8 *Trp53*^*−/−*^, ID8 *Trp53*^*−/−*^* Brca*^*−/−*^ and ID8 *Trp53*^*−/−*^* Brca2*^*−/−*^ cell lines were used to induce tumour development in mice to study the effects of olaparib on the survival of tumour-bearing mice. Olaparib was initially dissolved in DMSO and later in a mixture of PBS containing Captisol (MedChemExpress; HY-17031) to achieve final concentrations of 5% and 20%, respectively. The olaparib group received 18 daily IP injections of 50 mg/kg/day olaparib. The 48 mice (8 animals/group/cell line) were then monitored regularly and euthanized at a humane endpoint as indicated by any of the following: development of ascites evident by abdominal distension; piloerection; reduced mobility; and impaired blood flow to the limbs. The euthanasia was carried out by a short period of CO_2_ exposure followed by cervical dislocation. The mice were weighed, and necropsies were carried out to determine ascites volume, total tumour mass and spleen mass. The ascites was centrifuged at 2000 revolutions per minute (RPM) for 15 min to pellet the cells, and the supernatant was flash frozen. Tumours were fixed in 10% neutral buffered formalin (NBF) and later transferred to 70% ethanol.

#### The combination in vivo study

The ID8 *Trp53*^*−/−*^* Brca1*^*−/−*^ and ID8 *Trp53*^*−/−*^* Brca2*^*−/−*^ cell lines were used to induce tumour development in mice to study the effects of olaparib and anti-PD-L1 monotherapies and their combination on survival and TME composition. Olaparib was initially dissolved in DMSO and later in a mixture of PBS containing Captisol (MedChemExpress; HY-17031) to achieve final concentrations of 5% and 20%, respectively. The PD-L1 (Leinco Technologies Inc; P363) and isotype control (Leinco Technologies Inc; I-536) monoclonal antibodies were diluted in sterile PBS to produce the desired concentrations. The antibodies were administered in five 100 µL IP daily injections of 200 µg of antibody, followed by 100 µg doses every four days, for a total of 11 antibody injections (Supplementary Fig. S1).

Animals were randomly assigned to four groups for each of the two *Brca-*mutant cell lines: (1) olaparib, (2) anti-PD-L1, (3) combination and (4) the control group. All treatments began 25% into the expected survival period of the animals. Animals in the olaparib group received 18 daily IP injections of 50 mg/kg/day olaparib, as well as the appropriate dose of isotype control monoclonal antibody. The anti-PD-L1 group received 18 doses of the vehicle control for olaparib, (20% captisol, 5% DMSO in PBS); as well as the appropriate dose of the PD-L1 monoclonal antibody, depending on the day. The combination group received 18 daily injections of 50 mg/kg/day olaparib and the appropriate dose of PD-L1 monoclonal antibody. Finally, the control group received the vehicle control of olaparib as well as the isotype control antibody. The animals in this study were euthanized at two time points. To assess changes in TME composition in response to treatment, 40 mice (5 mice/cell line/treatment group) were euthanized 36 h after the last olaparib injections. To determine the effects of the treatments on survival, the remaining 64 mice (8 animals/group) were monitored on a daily basis and euthanized as described previously.

### Flow cytometry

#### Cells treated in vitro

Flow cytometry was used to assess the effects of olaparib treatment on PD-L1 expression in ID8 cells. The cells were seeded in a 6-well plate at a density of 6.0 × 10^4^ cells/ well and treated with 10 µM olaparib for 24 h. The negative control group received DMSO, and the positive control group was treated with recombinant 0.5 ng/ml mouse interferon gamma (IFN-γ; Peprotech, 315–05). Following treatment, the cells were washed with PBS and incubated in the Fixable Viability Stain (BD BioSciences; #564,406) in a 1:1000 dilution for 15 min at room temperature (RT). The cells were washed with PBS supplemented with 2% FBS and stained with the PD-L1-PE antibody (Bio Legend; #124,308) at a 1:200 dilution for 20 min at RT. The cells were once again washed with PBS + 2% FBS and fixed using 1% paraformaldehyde (PFA). The samples were stored in the dark at 4 °C overnight and data was acquired the following day using a CYTEK™ Aurora Spectral flow cytometer. The FlowJo program was used to determine the percentage of PD-L1 positive cells in each sample.

#### TME in vivo study

Peritoneal washes and spleen were collected from the mice 36 h after the end of treatment and processed as described previously [54]. In preparation for flow cytometry, T, B, and NK cells, as well as various myeloid cells were fixed and stained as described previously [54]. A list of antibodies included in each flow cytometry panel are provided in Supplementary Table S2.

### Immunofluorescence

Immunofluorescence staining for γH2AX was performed on ID8 cells treated with 10 µM olaparib for 24 h to determine the extent of DNA damage according to the previously described protocol [54], using Cell Signaling (2577L) antibody at a 1:200 dilution, incubated overnight. Images were acquired using the ZEISS Axioscope 5 Smart Laboratory Microscope (100 × objective) and quantification was performed using Orbit Image Analysis (percent positive cells).

### Immunohistochemistry

Immunohistochemical analysis of CD31 expression was performed on tumours collected at humane endpoint to determine the extent of vascularity in ID8 models. The method was performed as described previously [54], using an Abcam (ab28364) antibody at a 1:50 dilution for 2 h. Images were acquired using the Zeiss AxioScan Z1 (10X objective) and quantification was performed using Orbit Image Analysis (percent positive pixels).

### Cytokine array

Ascites supernatant was diluted in the LEGENDplex™ Cytokine Release Syndrome Panel assay buffer (BioLegend, #741,024). The staining was then carried out using the manufacturer’s protocol and flow cytometry was carried out using the BD LSR Fortessa flow cytometer on the same day. The data was analyzed using the LEGENDplex Quognit software (BioLegend).

### Analysis of RNA sequencing data

RNA-seq data from ID8 cell lines and IP tumours which were previously generated by the Vanderhyden lab [23] were used to assess transcriptomes related to PARP activity and differential TME gene expression. The cell lines were all of a similar passage number (< 20) and the IP tumours were collected at the humane endpoint. The R Studio program was used to create a normal distribution of the log-normalized RNA-seq values and the various sets of data were visualized using the “pheatmap” function in R. Pathway analyses were done by analysing the expression levels of genes which are associated with the pathway of interest (Supplementary Table S3).

### Statistical analysis

All statistical analyses were performed with Prism 9.0 (GraphPad Software Inc.) The Prism 9.0 software and R Studio were used to generate all the figures. Comparisons between two groups were performed using the student’s *t* test, and one-way analysis of variance (ANOVA) and Tukey’s multiple comparisons were used to compare three or more groups. The Log-Rank test was used to determine any significant differences in the survival data shown using Kaplan–Meier plots. Histogram data sets are presented as the means ± standard deviation (SD) or stranded error of the mean (SEM). Statistical significance was set at *p* ≤ 0.05 (*p ≤ 0.05; **p < 0.01; ***p < 0.001; ****p < 0.0001).

### Supplementary Information

**Additional file 1: Figure S1.** Treatment regimen in the second in vivo study. The mice were all injected with 5.0x10^6^ cells with a similar passage number (<10) by intraperitoneal injections on day zero. The treatments began 25% into the predicted survival period of each model. All treatments were provided using 100 μL intraperitoneal injections. All drugs were dissolved in sterile PBS. On days that the mice received both drugs (olaparib and the monoclonal antibody, or their controls), the drugs were administered using one 200 μL injection to reduce stress. In order to analyze the TME composition in response to treatment, 40 mice were collected 36 hours after the end treatment. The rest of the animals (*n*=64) which belonged to the survival group were collected at the humane endpoint to assess the impact of treatment on the survival of tumour-bearing mice.

**Additional file 2: Figure S2.** Gating strategy for the analysis of flow cytometry data. Peritoneal washes and spleens were collected and analyzed by flow cytometry approximately 36 hours after the end of therapy. (A) The gating strategy used to analyze the first flow cytometry panel is as follows: singlet, cell debris exclusion, live cell exclusion, leukocytes (CD45+), CD3+ (T cells), CD3- (B cells), DX5+ (natural killer cells). The T cell panel was further assessed using markers such as CD4, CD8, PD-1, LAG3, CD44, CD25, CD62L and TIGIT. (B) The gating strategy for the second panel included selection of singlets, cell debris exclusion, live cell exclusion, leukocytes (CD45+), CD3+ (T cells) and CD3- (Myeloid-like cells). The myeloid-like panel was further assessed by using markers for dendritic cells (DCs), myeloid-derived suppressor cells (MSDCs), monocytes, macrophages, and other myeloid-like cells. Fluorescence minus one (FMOs) represent the counter plots shown in each figure.

**Additional file 3: Figure S3.** Abundance of innate and adaptive immune cells within the ID8 *Trp53-/- Brca1-/- *spleens. Spleen tissues (*n*=5 mice per group) were collected from tumour-bearing mice injected with 5x10^6^ cells. The mice were treated with olaparib, anti-PD-L1 or their combination, and analyzed by flow cytometry approximately 36 hours after the end of treatment. Only the immune populations which are significantly different from isotype control or the combination therapy are shown. Each dot represents one biological replicate. Mean values with SD are shown. ISO indicates the isotype control group and OLA indicates the olaparib treated group. Analysis was done using a one-way ANOVA followed by a Tukey’s multiple comparison test. *p<0.05, **p<0.01, ***p<0.001.

**Additional file 4: Figure S4.** Abundance of innate and adaptive immune cells within the ID8 *Trp53-/- Brca2-/- *spleen. Spleen tissues (*n*=5 mice per group) were collected from tumour-bearing mice injected with 5x10^6^ cells. The mice were treated with olaparib, anti-PD-L1 or their combination, and analyzed by flow cytometry approximately 36 hours after the end of treatment. Only the immune populations which are significantly different from isotype control or the combination therapy are shown. Each dot represents one biological replicate. Mean values with SD are shown. ISO indicates the isotype control group and OLA indicates the olaparib treated group. Analysis was done using a one-way ANOVA followed by a Tukey’s multiple comparison test. *p<0.05, **p<0.01, ***p<0.001, ****p<0.0001.

**Additional file 5: Table S1.** Development timelines for various ID8 syngeneic models of HGSOC. The mice were each injected with 5.0x10^6^ cells at a similar passage number (<10) by intraperitoneal injection. Survival periods were calculated based on the mean survival of 8 mice bearing the same syngeneic tumour type in previous studies.

**Additional file 6: Table S2.** Fluorophore conjugated flow cytometry antibodies used to analyze the composition of the ovarian tumour microenvironment. All antibodies, with the exception of BV510, were diluted in PBS + 2% FBS. The viability BV510 stain was diluted in PBS only.

**Additional file 7: Table S3.** List of genes involved in the pathway analysis.

## Data Availability

RNA-seq data was accessed from GSE183368 as reported by Cook et al. [23].

## References

[1] Brett MR, Jennifer BP, Thomas AS, Brett MR, Jennifer BP, Thomas AS (2017). Epidemiology of ovarian cancer: a review. Cancer Biol Med.

[2] PDQ Adult Treatment Editorial Board. Ovarian Epithelial, Fallopian Tube, and Primary Peritoneal Cancer Treatment (PDQ®): Health Professional Version. 2023. In: PDQ Cancer Information Summaries. Bethesda (MD): National Cancer Institute (US); 2002–. PMID: 26389443. 26389443

[3] Timmermans M, Sonke GS, Van de Vijver KK, van der Aa MA, Kruitwagen RFPM (2018). No improvement in long-term survival for epithelial ovarian cancer patients: A population-based study between 1989 and 2014 in the Netherlands. Eur J Cancer.

[4] Radziszewska AU, Karczmarek-Borowska B, Wójcik S, Kluz T (2018). Survival rates among women with ovarian cancers diagnosed in the area of Podkarpacie province in the years 1990–2015. Contemp Oncol.

[5] Wright AA, Bohlke K, Armstrong DK, Bookman MA, Cliby WA, Coleman RL (2016). Neoadjuvant Chemotherapy for Newly Diagnosed, Advanced Ovarian Cancer: Society of Gynecologic Oncology and American Society of Clinical Oncology Clinical Practice Guideline. J Clin Oncol.

[6] Vergote I, Coens C, Nankivell M, Kristensen GB, Parmar MKB, Ehlen T (2018). Neoadjuvant chemotherapy versus debulking surgery in advanced tubo-ovarian cancers: pooled analysis of individual patient data from the EORTC 55971 and CHORUS trials. Lancet Oncol.

[7] Moore K, Colombo N, Scambia G, Kim BG, Oaknin A, Friedlander M (2018). Maintenance Olaparib in Patients with Newly Diagnosed Advanced Ovarian Cancer. N Engl J Med.

[8] Disilvestro P, Colombo N, Scambia G, Kim BG, Oaknin A, Friedlander M (2020). Efficacy of Maintenance Olaparib for Patients With Newly Diagnosed Advanced Ovarian Cancer With a BRCA Mutation: Subgroup Analysis Findings From the SOLO1 Trial. J Clin Oncol.

[9] Hall JM, Lee MK, Newman B, Morrow JE, Anderson LA, Huey B (1990). Linkage of Early-Onset Familial Breast Cancer to Chromosome 17q21. Science.

[10] Kwon Y, Rösner H, Zhao W, Selemenakis P, He Z, Kawale AS (2023). DNA binding and RAD51 engagement by the BRCA2 C-terminus orchestrate DNA repair and replication fork preservation. Nat Commun.

[11] Tarsounas M, Sung P (2020). The antitumorigenic roles of BRCA1–BARD1 in DNA repair and replication. Nat Rev Mol Cell Biol.

[12] Prakash R, Zhang Y, Feng W, Jasin M (2015). Homologous Recombination and Human Health: The Roles of BRCA1, BRCA2, and Associated Proteins. Cold Spring Harb Perspect Biol.

[13] Roy R, Chun J, Powell SN (2012). BRCA1 and BRCA2: different roles in a common pathway of genome protection. Nat Rev Cancer.

[14] Wen WX, Leong CO (2019). Association of BRCA1- and BRCA2-deficiency with mutation burden, expression of PD-L1/PD-1, immune infiltrates, and T cell-inflamed signature in breast cancer. PLoS ONE.

[15] Kim DS, Challa S, Jones A, Kraus WL (2020). PARPs and ADP-ribosylation in RNA biology: from RNA expression and processing to protein translation and proteostasis. Genes Dev.

[16] DiSilvestro P, Colombo N, Scambia G, Kim BG, Oaknin A, Friedlander M (2020). Efficacy of Maintenance Olaparib for Patients With Newly Diagnosed Advanced Ovarian Cancer With a BRCA Mutation: Subgroup Analysis Findings From the SOLO1 Trial. J Clin Oncol.

[17] Kuchenbaecker KB, Hopper JL, Barnes DR, Phillips KA, Mooij TM, Roos-Blom MJ (2017). Risks of Breast, Ovarian, and Contralateral Breast Cancer for BRCA1 and BRCA2 Mutation Carriers. JAMA.

[18] Bolton KL (2012). Association Between BRCA1 and BRCA2 Mutations and Survival in Women With Invasive Epithelial Ovarian Cancer. JAMA.

[19] Wen WX, Leong CO (2019). Association of BRCA1- And BRCA2-deficiency with mutation burden, expression of PD-L1/ PD-1, immune infiltrates, and T cell-inflamed signature in breast cancer. PLoS One..

[20] Wei Y, Ou T, Lu Y, Wu G, Long Y, Pan X (2020). Classification of ovarian cancer associated with BRCA1 mutations, immune checkpoints, and tumor microenvironment based on immunogenomic profiling. PeerJ.

[21] Walton JB, Farquharson M, Mason S, Port J, Kruspig B, Dowson S (2017). CRISPR/Cas9-derived models of ovarian high grade serous carcinoma targeting Brca1, Pten and Nf1, and correlation with platinum sensitivity. Sci Rep.

[22] Walton J, Blagih J, Ennis D, Leung E, Dowson S, Farquharson M (2016). CRISPR/Cas9-mediated Trp53 and Brca2 knockout to generate improved murine models of ovarian high-grade serous carcinoma. Cancer Res.

[23] Cook DP, Galpin KJC, Rodriguez GM, Shakfa N, Wilson-Sanchez J, Echaibi M (2023). Comparative analysis of syngeneic mouse models of high-grade serous ovarian cancer. Commun Biol..

[24] Meng J, Peng J, Feng J, Maurer J, Li X, Li Y (2021). Niraparib exhibits a synergistic anti-tumor effect with PD-L1 blockade by inducing an immune response in ovarian cancer. J Transl Med..

[25] Bunting K, Rao S, Hardy K, Woltring D, Denyer GS, Wang J (2007). Genome-Wide Analysis of Gene Expression in T Cells to Identify Targets of the NF-κB Transcription Factor c-Rel. J Immunol.

[26] Bao T, Liu J, Leng J, Cai L (2021). The cGAS–STING pathway: more than fighting against viruses and cancer. Cell and Bioscience.

[27] Li CW, Lim SO, Xia W, Lee HH, Chan LC, Kuo CW (2016). Glycosylation and stabilization of programmed death ligand-1 suppresses T-cell activity. Nat Commun.

[28] Jiao S, Xia W, Yamaguchi H, Wei Y, Chen MK, Hsu JM (2017). PARP inhibitor upregulates PD-L1 expression and enhances cancer-associated immunosuppression. Clin Cancer Res.

[29] Bruand M, Barras D, Mina M, Ghisoni E, Morotti M, Lanitis E (2021). Cell-autonomous inflammation of BRCA1-deficient ovarian cancers drives both tumor-intrinsic immunoreactivity and immune resistance via STING. Cell Rep.

[30] Sato E, Olson SH, Ahn J, Bundy B, Nishikawa H, Qian F (2005). Intraepithelial CD8 ^+^ tumor-infiltrating lymphocytes and a high CD8 ^+^ /regulatory T cell ratio are associated with favorable prognosis in ovarian cancer. Proc Natl Acad Sci.

[31] Zhang L, Conejo-Garcia JR, Katsaros D, Gimotty PA, Massobrio M, Regnani G (2003). Intratumoral T Cells, Recurrence, and Survival in Epithelial Ovarian Cancer. N Engl J Med.

[32] Lieber S, Reinartz S, Raifer H, Finkernagel F, Dreyer T, Bronger H (2018). Prognosis of ovarian cancer is associated with effector memory CD8 ^+^ T cell accumulation in ascites, CXCL9 levels and activation-triggered signal transduction in T cells. Oncoimmunology.

[33] Kalia V, Sarkar S (2018). Regulation of Effector and Memory CD8 T Cell Differentiation by IL-2—A Balancing Act. Front Immunol.

[34] Lai YP, Lin CC, Liao WJ, Tang CY, Chen SC (2009). CD4+ T Cell-Derived IL-2 Signals during Early Priming Advances Primary CD8+ T Cell Responses. PLoS One.

[35] Toumi R, Yuzefpolskiy Y, Vegaraju A, Xiao H, Smith KA, Sarkar S (2022). Autocrine and paracrine IL-2 signals collaborate to regulate distinct phases of CD8 T cell memory. Cell Rep.

[36] Preston CC, Maurer MJ, Oberg AL, Visscher DW, Kalli KR, Hartmann LC (2013). The Ratios of CD8+ T Cells to CD4+CD25+ FOXP3+ and FOXP3- T Cells Correlate with Poor Clinical Outcome in Human Serous Ovarian Cancer. PLoS One.

[37] Waki K, Kawano K, Tsuda N, Komatsu N, Yamada A (2020). CD4/CD8 ratio is a prognostic factor in IgG nonresponders among peptide vaccine-treated ovarian cancer patients. Cancer Sci.

[38] Brodbeck T, Nehmann N, Bethge A, Wedemann G, Schumacher U (2014). Perforin-dependent direct cytotoxicity in natural killer cells induces considerable knockdown of spontaneous lung metastases and computer modelling-proven tumor cell dormancy in a HT29 human colon cancer xenograft mouse model. Mol Cancer.

[39] Paul S, Lal G (2017). The Molecular Mechanism of Natural Killer Cells Function and Its Importance in Cancer Immunotherapy. Front Immunol.

[40] Liu Y, Cheng Y, Xu Y, Wang Z, Du X, Li C (2017). Increased expression of programmed cell death protein 1 on NK cells inhibits NK-cell-mediated anti-tumor function and indicates poor prognosis in digestive cancers. Oncogene.

[41] Penn CA, Lester J, Bohrer K, Moon C, Yearley J, Karlan BY (2019). PD-1/PD-L1 expression in mutated ovarian cancers. Gynecol Oncol.

[42] Kornepati AVR, Boyd JT, Murray CE, Saifetiarova J, la Peña Avalos de Rogers BCM (2022). Tumor Intrinsic PD-L1 Promotes DNA Repair in Distinct Cancers and Suppresses PARP Inhibitor-Induced Synthetic Lethality. Cancer Res.

[43] Dai Y, Sun C, Feng Y, Jia Q, Zhu B (2018). Potent immunogenicity in BRCA1-mutated patients with high-grade serous ovarian carcinoma. J Cell Mol Med.

[44] Strome SE, Dong H, Tamura H, Voss SG, Flies DB, Tamada K (2003). B7–H1 blockade augments adoptive T-cell immunotherapy for squamous cell carcinoma. Cancer Res.

[45] Pauken KE, Wherry EJ (2015). Overcoming T cell exhaustion in infection and cancer. Trends Immunol.

[46] Kawai H, Li H, Chun P, Avraham S, Avraham HK (2002). Direct interaction between BRCA1 and the estrogen receptor regulates vascular endothelial growth factor (VEGF) transcription and secretion in breast cancer cells. Oncogene.

[47] Wang L, Di LJ (2014). BRCA1 And Estrogen/Estrogen Receptor In Breast Cancer: Where They Interact?. Int J Biol Sci.

[48] Baaten BJG, Tinoco R, Chen AT, Bradley LM (2012). Regulation of Antigen-Experienced T Cells: Lessons from the Quintessential Memory Marker CD44. Front Immunol..

[49] Huang RY, Francois A, McGray AR, Miliotto A, Odunsi K (2017). Compensatory upregulation of PD-1, LAG-3, and CTLA-4 limits the efficacy of single-agent checkpoint blockade in metastatic ovarian cancer. Oncoimmunology.

[50] Koyama S, Akbay EA, Li YY, Herter-Sprie GS, Buczkowski KA, Richards WG (2016). Adaptive resistance to therapeutic PD-1 blockade is associated with upregulation of alternative immune checkpoints. Nat Commun.

[51] Miller BC, Sen DR, Al Abosy R, Bi K, Virkud YV, LaFleur MW (2019). Subsets of exhausted CD8+ T cells differentially mediate tumor control and respond to checkpoint blockade. Nat Immunol.

[52] Hodgins JJ, Abou-Hamad J, Hagerman A, Yakubovich E, de TaneseSouza C, Marotel M (2022). More than a ligand: PD-L1 promotes oncolytic virus infection via a metabolic shift that inhibits the type I interferon pathway. bioRxiv.

[53] Di DJ, Gulen MF, Saidoune F, Thacker VV, Yatim A, Sharma K (2022). The cGAS–STING pathway drives type I IFN immunopathology in COVID-19. Nature.

[54] Rodriguez GM, Galpin KJC, Cook DP, Yakubovich E, Maranda V, Macdonald EA (2022). The Tumor Immune Profile of Murine Ovarian Cancer Models: An Essential Tool for Ovarian Cancer Immunotherapy Research. Cancer Res Commun.

