# Supplementary material for: BRCA1 and BRCA2 deficient tumour models generate distinct ovarian tumour microenvironments and differential responses to therapy
Source: J Ovarian Res. 2023 Nov 28;16:231. doi: 10.1186/s13048-023-01313-z (PMC10683289; doi:10.1186/s13048-023-01313-z)
Supplement: Supplementary file 1 — Additional file 1: Figure S1. Treatment regimen in the second in vivo study. The mice were all injected with 5.0x10^6^ cells with a similar passage number (<10) by intraperitoneal injections on day zero. The treatments began 25% into the predicted survival period of each model. All treatments were provided using 100 μL intraperitoneal injections. All drugs were dissolved in sterile PBS. On days that the mice received both drugs (olaparib and the monoclonal antibody, or their controls), the drugs were administered using one 200 μL injection to reduce stress. In order to analyze the TME composition in response to treatment, 40 mice were collected 36 hours after the end treatment. The rest of the animals (n=64) which belonged to the survival group were collected at the humane endpoint to assess the impact of treatment on the survival of tumour-bearing mice. [file 13048_2023_1313_MOESM1_ESM.pdf]

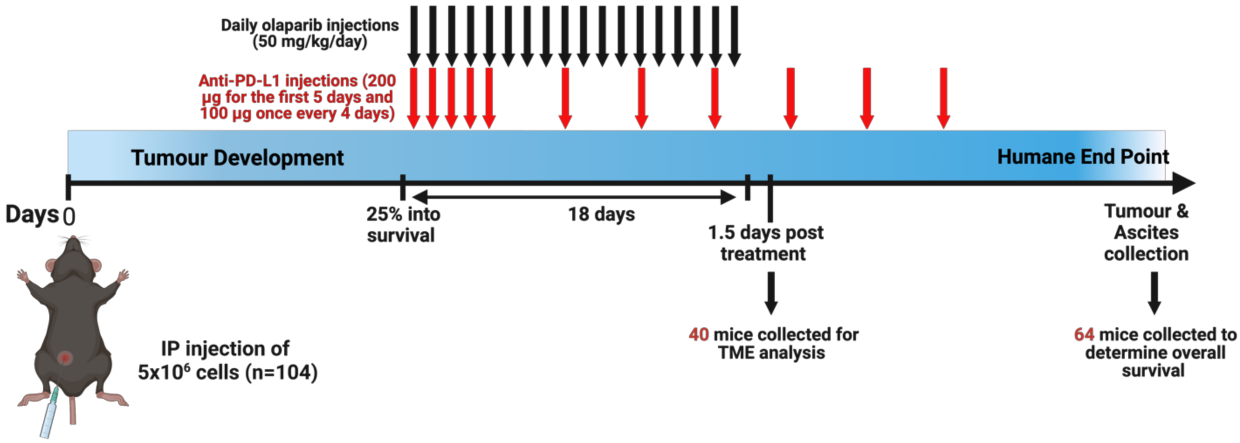

Figure S1. Treatment regimen in the second *in vivo* study. The mice were all injected with 5.0×10^6 cells with a similar passage number (<10) by intraperitoneal injections on day zero. The treatments began 25% into the predicted survival period of each model. All treatments were provided using 100 µL intraperitoneal injections. All drugs were dissolved in sterile PBS. On days that the mice received both drugs (olaparib and the monoclonal antibody, or their controls), the drugs were administered using one 200 µL injection to reduce stress. In order to analyze the TME composition in response to treatment, 40 mice were collected 36 hours after the end treatment. The rest of the animals (n=64) which belonged to the survival group were collected at the humane endpoint to assess the impact of treatment on the survival of tumour-bearing mice.
