# Supplementary material for: BRCA1 and BRCA2 deficient tumour models generate distinct ovarian tumour microenvironments and differential responses to therapy
Source: J Ovarian Res. 2023 Nov 28;16:231. doi: 10.1186/s13048-023-01313-z (PMC10683289; doi:10.1186/s13048-023-01313-z)
Supplement: Supplementary file 2 — Additional file 2: Figure S2. Gating strategy for the analysis of flow cytometry data. Peritoneal washes and spleens were collected and analyzed by flow cytometry approximately 36 hours after the end of therapy. (A) The gating strategy used to analyze the first flow cytometry panel is as follows: singlet, cell debris exclusion, live cell exclusion, leukocytes (CD45+), CD3+ (T cells), CD3- (B cells), DX5+ (natural killer cells). The T cell panel was further assessed using markers such as CD4, CD8, PD-1, LAG3, CD44, CD25, CD62L and TIGIT. (B) The gating strategy for the second panel included selection of singlets, cell debris exclusion, live cell exclusion, leukocytes (CD45+), CD3+ (T cells) and CD3- (Myeloid-like cells). The myeloid-like panel was further assessed by using markers for dendritic cells (DCs), myeloid-derived suppressor cells (MSDCs), monocytes, macrophages, and other myeloid-like cells. Fluorescence minus one (FMOs) represent the counter plots shown in each figure. [file 13048_2023_1313_MOESM2_ESM.pdf]

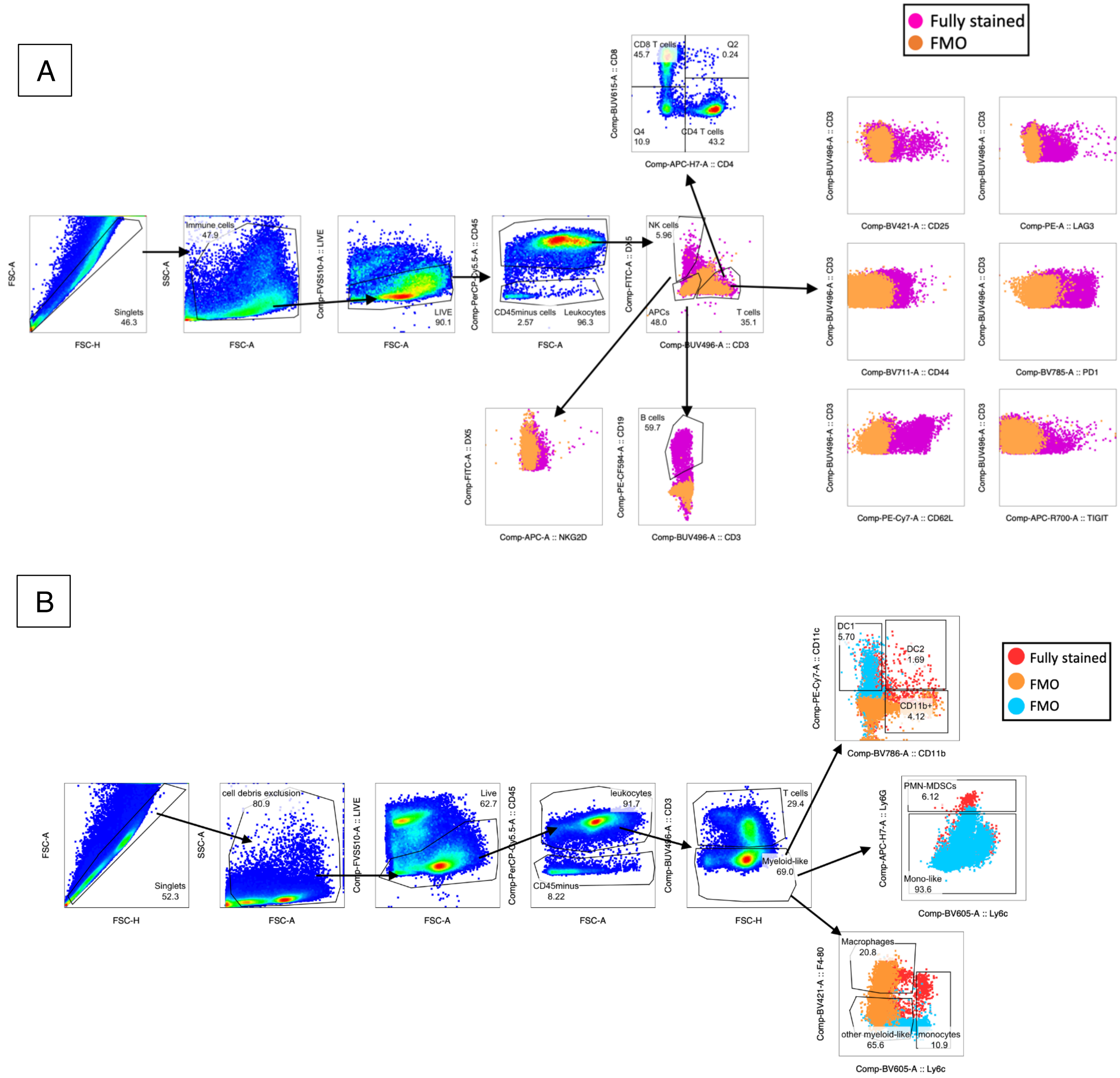

Figure S2. Gating strategy for the analysis of flow cytometry data. Peritoneal washes and spleens were collected and analyzed by flow cytometry approximately 36 hours after the end of therapy. (A) The gating strategy used to analyze the first flow cytometry panel is as follows: singlet, cell debris exclusion, live cell exclusion, leukocytes (CD45+), CD3+ (T cells), CD3- (B cells), DX5+ (natural killer cells). The T cell panel was further assessed using markers such as CD4, CD8, PD-1, LAG3, CD44, CD25, CD62L and TIGIT. (B) The gating strategy for the second panel included selection of singlets, cell debris exclusion, live cell exclusion, leukocytes (CD45+), CD3+ (T cells) and CD3- (Myeloid-like cells). The myeloid-like panel was further assessed by using markers for dendritic cells (DCs), myeloid-derived suppressor cells (MSDCs), monocytes, macrophages, and other myeloid-like cells. Fluorescence minus one (FMOs) represent the counter plots shown in each figure.
