# Supplementary material for: BRCA1 and BRCA2 deficient tumour models generate distinct ovarian tumour microenvironments and differential responses to therapy
Source: J Ovarian Res. 2023 Nov 28;16:231. doi: 10.1186/s13048-023-01313-z (PMC10683289; doi:10.1186/s13048-023-01313-z)
Supplement: Supplementary file 3 — Additional file 3: Figure S3. Abundance of innate and adaptive immune cells within the ID8 Trp53-/- Brca1-/- spleens. Spleen tissues (n=5 mice per group) were collected from tumour-bearing mice injected with 5x10^6^ cells. The mice were treated with olaparib, anti-PD-L1 or their combination, and analyzed by flow cytometry approximately 36 hours after the end of treatment. Only the immune populations which are significantly different from isotype control or the combination therapy are shown. Each dot represents one biological replicate. Mean values with SD are shown. ISO indicates the isotype control group and OLA indicates the olaparib treated group. Analysis was done using a one-way ANOVA followed by a Tukey’s multiple comparison test. *p<0.05, **p<0.01, ***p<0.001. [file 13048_2023_1313_MOESM3_ESM.pdf]

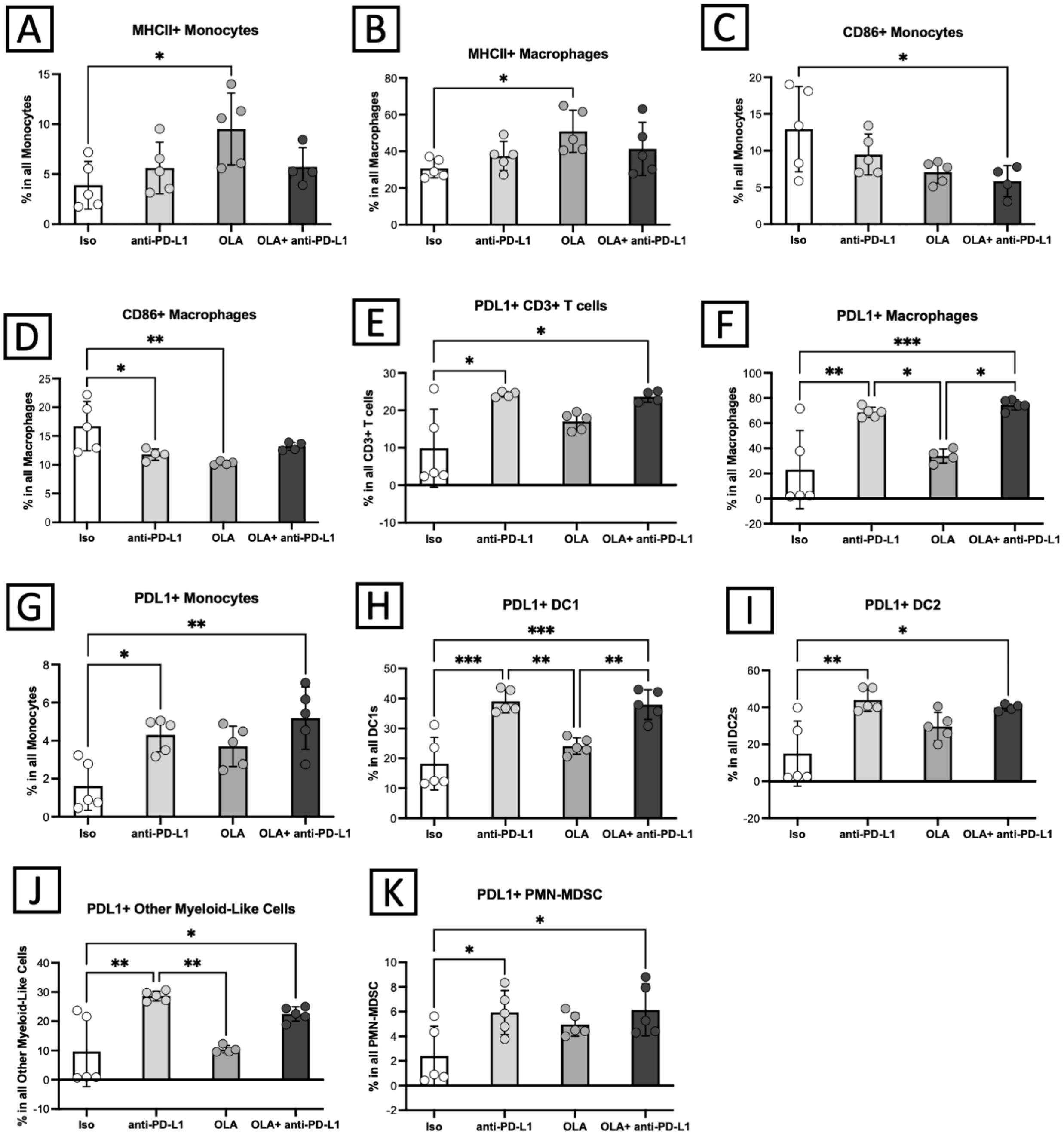

Figure S3. Abundance of innate and adaptive immune cells within the ID8 *Trp53$^{-/-}$ Brca1$^{-/-}$* spleens. Spleen tissues (n=5 mice per group) were collected from tumour-bearing mice injected with $5x10^6$ cells. The mice were treated with olaparib, anti-PD-L1 or their combination, and analyzed by flow cytometry approximately 36 hours after the end of treatment. Only the immune populations which are significantly different from isotype control or the combination therapy are shown. Each dot represents one biological replicate. Mean values with SD are shown. ISO indicates the isotype control group and OLA indicates the olaparib treated group. Analysis was done using a one-way ANOVA followed by a Tukey's multiple comparison test. *p<0.05, **p<0.01, ***p<0.001.
